# Quantification of left ventricular deformation fields from undersampled radial, real-time cardiac MRI

**DOI:** 10.1186/1532-429X-16-S1-P366

**Published:** 2014-01-16

**Authors:** Francisco Contijoch, Kelly Rogers, Brian Avants, Paul Yushkevich, Vahid Hoshmand, Robert C Gorman, Yuchi Han, Walter R Witschey

**Affiliations:** 1 University of Pennyslvania, Philadelphia, Pennsylvania, USA

## Background

Regional ventricular elasticity is altered in ischemic heart disease and LV hypertrophy, potentially contributing to and sustaining a ventricular remodeling process. Conventional methods to measure regional ventricular elasticity in preclinical animal models require high fidelity ventricular pressure measurement, sonomicrometry transducers and dynamic modulation of preload. These procedures are highly invasive and provide only limited regional information; patient studies are prohibitive. We dynamically measure global LV elastance using undersampled radial real-time MRI and seek to find regional LV elastance in these studies. This requires tracking LV motion derived from wall thickness measurements in a large number of real-time image frames at high accuracy. We assessed several motion tracking algorithms and characterize their fidelity with respect to true motion labeled by experts and sensitivity to residual radial streak artifact.

## Methods

A series of short axis MR slices were acquired in 9 patients using a golden-angle radial bSSFP trajectory (total views = 4000, radial views per image = 34, shared profiles = 31, reconstructed frame rate = 98 fps) and reconstructed via conjugate gradient-SENSE based iterative reconstruction. Two independent researchers manually segmented the datasets and the movies were restricted to three full heartbeats to limit the number of images. Image registrations were performed between the initial end-diastolic frame and all subsequent frames. The image registrations were performed using b-spline and symmetric diffeomorphic (SyN) image registration across a range of spatial scales (shown in Table 1). Four separate image registration metrics were investigated (mutual information, mean-squared overlap, cross-correlation, and demons). Although we expected mean squares and cross-correlation to perform better in this intramodality registration, radial streaking could potentially corrupt data integrity. The resulting deformation fields were evaluated using an array of metrics (dice coefficient, area overlap, false positive, and false negative rate) for all registrations, image frames and patients.

**Table 1:** 

Transformation:			Metric:
**Transform:**	**Grid Size:**	**Step Size:**	

BSplineSyN or SyN	32 × 32 (6.88 mm × 6.88 mm)	0.1	Mutual Information

		1.0	Mean Squres

		1.8	Cross Correlation (radius = 8)

	16 × 16 (13.75 mm × 13.75 mm)	0.1	Cross Correlation (radius = 32)

		1.0	Demons

	8 × 8 (27.5 mm × 27.5 mm)	0.1	

		1.0	

## Results

The results obtained using 70 different registrations on 9 patients illustrate symmetric diffeomorphic registrations outperformed b-spine, an intermediate spatial scale of 13.75 mm × 13.75 mm outperformed larger and smaller grids, and the larger step-size (1.0) improved results relative to the smaller step size (0.1). We believe the ideal spatial scale is a tradeoff between sufficient resolution to capture the cardiac motion and avoiding local minimums. In terms of metrics, smaller changes in the results were observed but the mean squares resulted in the best overall result.

## Conclusions

Symmetric diffeomorphic image registration of moderate spatial scale allows for accurate tracking of myocardial motion and estimation of wall thickness from a large number of real-time image frames.

## Funding

K99-HL108157, R01-HL103723, T32HL007954, T32-EB009384.

**Figure 1:**
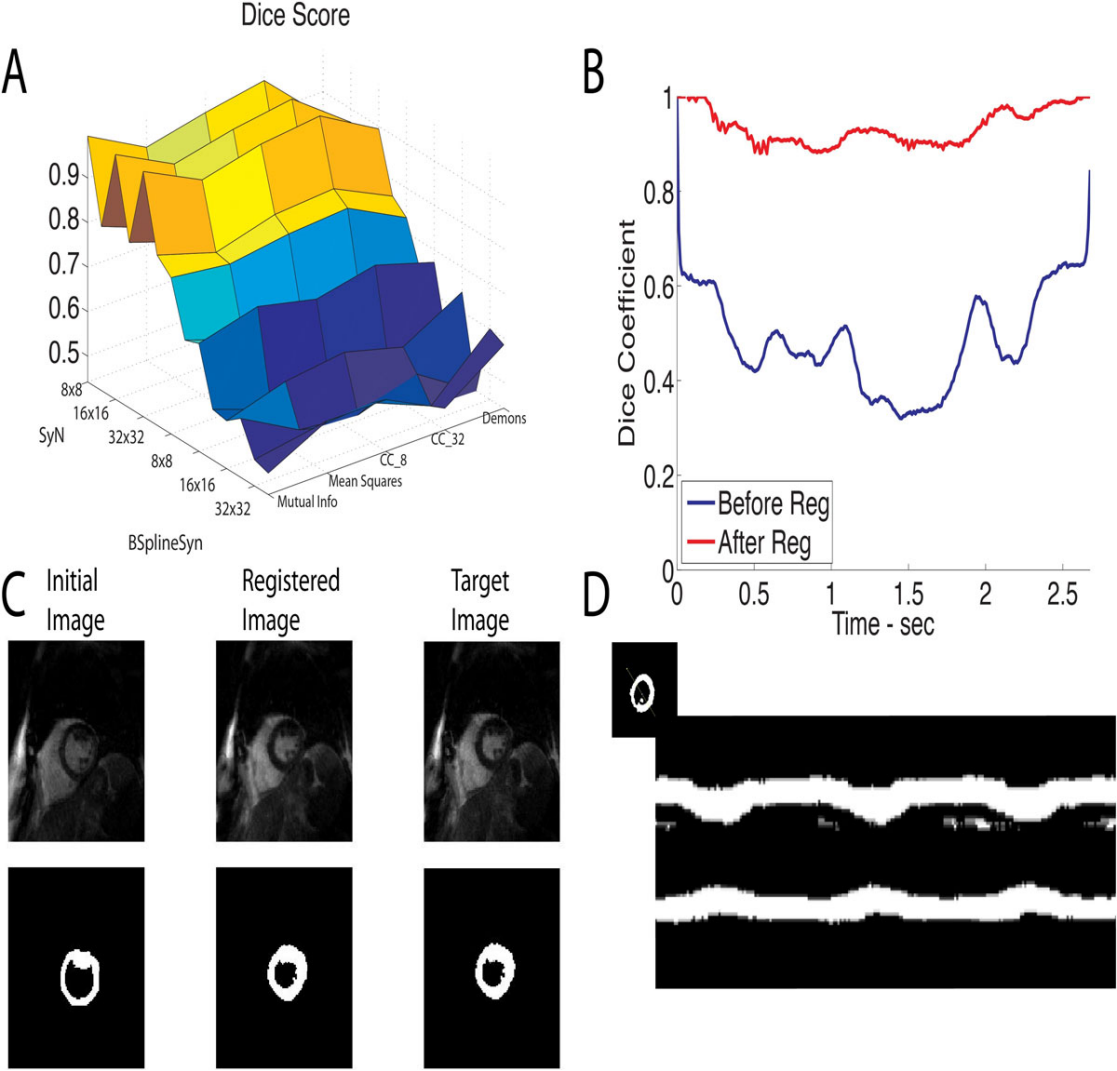
**A) The average Dice score across all registrations, all frames (and both manual segmentations) is shown in the top left**. A range of image metrics and registration algorithms were employed to identify an optimal combination. B) The Dice coefficient over the three heart beats is shown prior to (blue) and after registration (red). This result is obtained using the best registration identified across all 9 patients. C) The success of the registration is illustrated with the underlying images and transforms masks. The frame shown corresponds to the worst Dice score prior to registration. As you can see, both the transformated image and mask show close agreement. D) We are interested in measuring wall thickness. Here we illustrate how this algorithm allows us to observe the underlying three heartbeats by showing a cross section of the myocardial mask.

